# Deceptive appearance of primary mucinous carcinoma of the eyelid: A rare case and literature review

**DOI:** 10.22336/rjo.2025.48

**Published:** 2025

**Authors:** Sonali Vinay Kumar, Manoj Gopal Madakshira, Vinay Kumar, Sanjay Kumar Mishra, Alok Sati, Natasha Vinay Kumar

**Affiliations:** 1 Department of Ophthalmology, Command Hospital Eastern Command, Kolkata, West Bengal, India; 2 Department of Pathology, Command Hospital Eastern Command, Kolkata, West Bengal, India; 3 Department of Anatomy, JIS School of Medical Science and Research, Howrah, West Bengal, India; 4 Department of Ophthalmology, Army Hospital Research and Referral, Delhi, India; 5471 Field Hospital, Arunachal Pradesh, India; 6 Department of Medicine, Sri Devaraj Urs Medical College, Kolar, Karnataka, India

**Keywords:** primary mucinous carcinoma, eyelid, rare case, misdiagnosis, wide local excision, PMC = Primary mucinous carcinoma, WLE = wide local incision, HPE = histopathological examination, PET = positron emission tomography, MMS = Mohs micrographic surgery

## Abstract

Primary mucinous carcinoma (PMC) of the eyelid is an exceedingly rare malignancy that often presents as an innocuous, slow-growing lesion, leading to frequent misdiagnosis as a benign entity. This diagnostic challenge can lead to delayed treatment and potential recurrence. We report a unique case of PMC in a patient who presented with a clinically benign-appearing lesion, initially considered for differential diagnoses such as keratoacanthoma, epidermoid cyst, and pilomatrixoma. A diagnostic surprise emerged when histopathological evaluation confirmed PMC. Despite recommendations for wide local excision, the patient opted for observation due to concerns about postoperative morbidity. Over a two-year follow-up period, no recurrence was noted. This case highlights the deceptive presentation of PMC, the importance of histopathological confirmation, and the role of individualized patient management. A review of the literature further underscores the rarity of this entity and the necessity for long-term surveillance.

## Introduction

Primary mucinous carcinoma (PMC) is a rare adnexal tumor arising from the eccrine sweat gland. It is a highly uncommon entity, with only about 300 cases reported in the literature since its initial documentation by Lennox et al. in 1952, and was first described as mucinous adenocystic carcinoma by Mendoza et al. [1,2]. It accounts for less than 0.1% of all eyelid malignancies. Its subtle and unassuming clinical presentation often results in diagnostic delays, with many cases being incidentally identified on histopathological examination. This entity is frequently misdiagnosed due to its slow growth and benign appearance. The lesion presents as a slow-growing, painless mass. It can easily be mistaken for a benign lesion such as a chalazion, epidermoid cyst, or hemangioma, leading to a delay in diagnosis and treatment [3,4]. They commonly arise in the head and neck region, with the eyelid being the most common site of origin, accounting for 41% [5]. This entity is more commonly observed in males and most frequently appears between the ages of 50 and 70 years [6]. The prevalence is higher in white patients compared to Asian and African Americans. Its differentiation from metastatic mucinous carcinoma, particularly from the gastrointestinal tract or breast, is critical for appropriate management [4,6]. Histopathological examination is essential for definitive diagnosis, as clinical and radiological features alone may not distinguish it from other benign or malignant eyelid tumors—immunohistochemical markers such as CK 7 positivity and CK 20 negativity help in confirming its primary adnexal origin. The challenge in managing PMC lies not only in its elusive nature but also in balancing oncologic safety with functional preservation, particularly in cases where surgical excision poses the risk of anatomical disruption. Herein, we report a case where a patient, following complete surgical excision of an eyelid lesion, was unexpectedly diagnosed with PMC and faced the difficult choice between re-excision and close surveillance. This case highlights the diagnostic and therapeutic challenges associated with this rare malignancy. A review of the literature was also provided to emphasize the importance of early recognition and appropriate surgical intervention to prevent recurrence.

## Case report

**Fig. 1:**
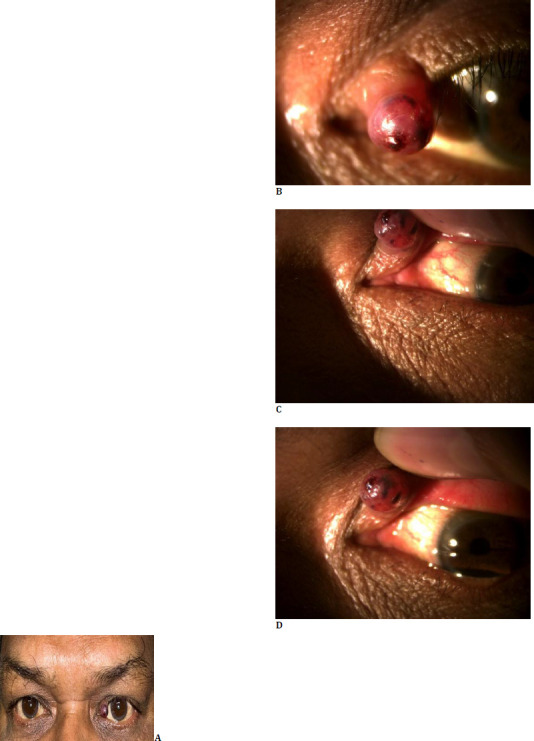
**A-D** Clinical photograph depicting a nodular lesion on the medial aspect of the left upper eyelid, with the punctum and posterior lamella unaffected

A 40-year-old male patient presented with a history of a slowly enlarging, painless lesion over the medial part of the left upper eyelid for the past 3 months. The patient did not give a history of blurring of vision/trauma/discharge from the eye, pain, double vision, or prior ocular intervention. The patient’s personal and family history were not significant. On clinical examination, the best corrected vision in both eyes was 6/6. Pupils were round, central, reacting to light. Intraocular pressure with the non-contact tonometer was 20 mm in the right eye and 18 mm in the left eye. Anterior and posterior segment evaluation in the right eye was remarkable. Left eye examination showed a lesion over the medial part of the left upper eyelid, about 5 mm x 5 mm in size, nodular, firm, non-tender, reddish in color, well circumscribed. The surface of the lesion was smooth and non-ulcerated and associated with a central punctum. The lesion was found arising from the eyelid margin, and no discharge was observed from it (**Fig. 1A, B**). The punctum was spared, with no loss of eyelashes, lid architecture distortion, or posterior lamella involvement, and no sign of telangiectasia (**Fig. 1C, D**).

**Fig. 2:**
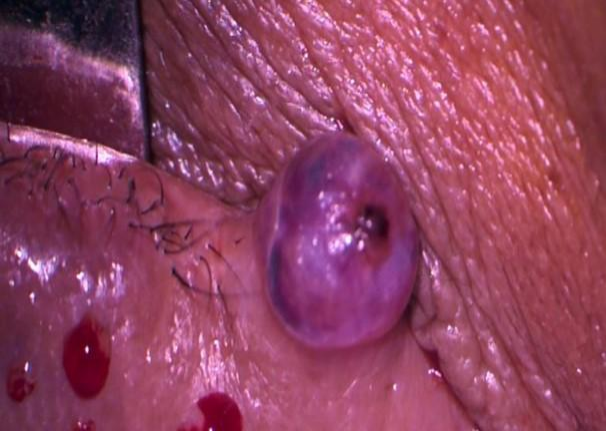
Intraoperative view of the lesion

There was no evidence of localized and generalized lymphadenopathy. Keratoacanthoma, epidermoid cyst, and pilomatrixoma were considered as differential diagnoses based on clinical features. Since no sign of malignancy was present, only benign lesions were considered. All routine hematological and biochemical tests were within normal limits. Under the impression that the lesion was benign, we carried out an excision biopsy, though a wide local excision (WLE) was not attempted at this time. The lesion was excised in toto under local anesthesia, and the base of the lesion was cauterized after excision (**Fig. 2**).

**Fig. 3:**
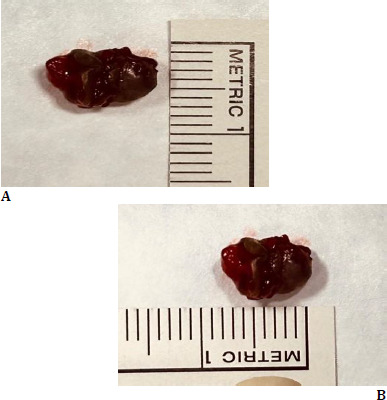
**A, B** Clinical image displaying the excised specimen (5 x 5 mm in size)

The size of the lesion was 5 mm x 5 mm, and it was sent for histopathological examination (HPE) (**Fig. 3A, B**). The wound was closed with 5-0 vicryl. The patient was found to be in stable condition following surgery and was started on oral antibiotics and anti-inflammatory drugs.

**Fig. 4:**
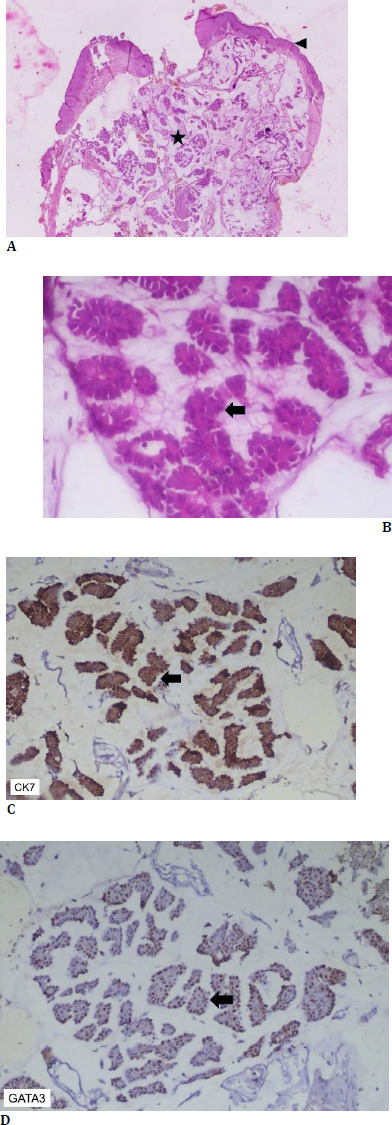
**A** Hematoxylin and Eosin stain (1 x magnification) shows tissue biopsy lined by stratified squamous epithelium (arrowhead) with underlying invasive tumor (star); **B**. Hematoxylin and Eosin stain (40 x magnification) shows an invasive tumor arranged in the form of small islands (arrow), which are seen to float in abundant extracellular mucin; **C**. CK7 Immunohistochemistry (20 x magnification) shows cytoplasmic expression in the tumor cells (arrow); **D**. GATA3 Immunohistochemistry (20 x magnification) shows nuclear expression in the tumor cells (arrow)

Histopathological examination showed moderate acanthosis with basket-weave keratin. The dermis showed a poorly circumscribed lesion composed of an invasive tumor arranged in the form of glands, micro papillae, and islands, which were observed to be floating in extensive extracellular mucin. The tumor cells were polygonal, had a high N: C ratio, open chromatin, conspicuous basophilic nucleoli, and a moderate amount of amphophilic cytoplasm (**Fig. 4A, B**). Immunohistochemistry showed the tumor cells to be positive for CK 7 (cytoplasmic) and GATA3 (nuclear) while being negative for CK20, p63, CDX2, and TTF1 (**Fig. 4C, D**).

**Fig. 5:**
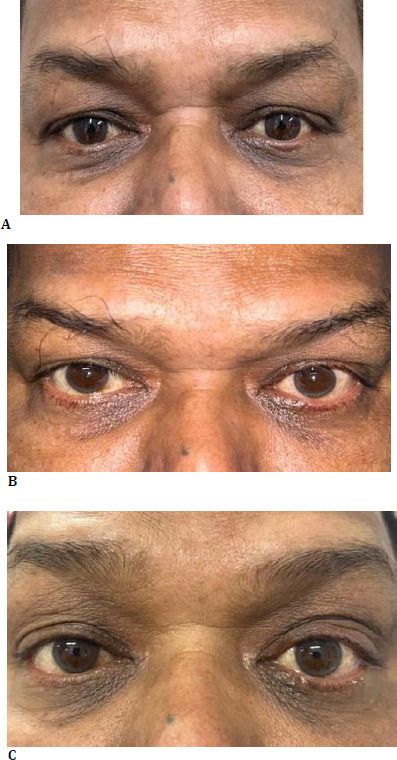
**A-C** Postoperative appearance at one week, one year, and two years after excision, showing no signs of recurrence

The observed features strongly suggested primary mucinous carcinoma of the eyelid skin. A positron emission tomography (PET) scan was advised, which did not show any sign of metastasis. The patient was advised to undergo a wide surgical excision with lid reconstruction to minimize the risk of recurrence. However, the patient declined further surgery and opted for observation, as he remained asymptomatic following the initial procedure. He agreed to attend monthly OPD visits and consent to surgery if any symptoms developed. The patient was monitored for 2 years post-surgery, during which no signs of recurrence were detected, and he did not report any symptoms (**Fig. 5A-C**).

## Discussion

Primary mucinous carcinoma of the eyelid typically follows a benign clinical course, with lymph node or distant metastasis being uncommon. The reported incidence of regional metastasis is approximately 10%. The tumor’s indolent nature is attributed to its uncontrolled mucin production, which impairs cellular nutrition, leading to reduced cancer cell growth and differentiation.

Primary mucinous carcinoma of the eyelid is a low-grade malignancy with high local recurrence rates (up to 40%) if not excised with clear margins. The mainstay of treatment in this entity is wide local excision with at least 10 mm margins or Mohs micrographic surgery (MMS) to achieve histologically negative margins. Mohs micrographic surgery has been advocated as an optimal approach for margin control while preserving uninvolved tissue. However, in scenarios where surgical morbidity is a significant concern, tailored management strategies must be considered. This tumor has been infrequently observed in Indian patients, with limited reports available in the literature (**Table 1**) [7,8].

**Table 1:** Summary of documented cases of primary mucinous carcinoma of the eyelid in Indian patients

Report	Follow-up	Patient profile	Presentation	Treatment
Krishnakumar et al.	No recurrence (2004) (10 months)	40-year-old male	Swelling of the right upper lid	Excision with lid reconstruction
Chauhan et al.	No recurrence (2009)	62-year-old male	Nodular left lower lid lesion	Excision with lid reconstruction
Khumanthem et al.	No recurrence (2024) (6 months)	64-year-old male	Left upper eyelid polypoidal lesion	Excision with lid reconstruction

To date, our case stands as the fourth documented occurrence of primary mucinous carcinoma of the eyelid in the Indian subcontinent. In our case, the early presentation was deceptive. The patient initially exhibited a seemingly benign growth on the eyelid, raising no immediate suspicion of malignancy. This prompted consideration of differential diagnosis, such as keratoacanthoma, epidermoid cyst, and pilomatrixoma. However, histopathological analysis unveiled an unexpected diagnosis of primary mucinous carcinoma of the eyelid, a rare and often misdiagnosed entity. This emphasizes the necessity of biopsy even for seemingly harmless eyelid lesions. Furthermore, existing literature frequently describes cases where wide local excision (WLE) with at least a 10 mm margin is the treatment of choice in this entity to ensure complete removal and prevent recurrence. Studies, such as those by Marra et al., Chavez et al., Baker et al., and Tak et al., emphasize the role of aggressive surgical management, with some advocating sentinel lymph node biopsy to rule out regional spread [9-12].

The diagnostic revelation was a turning point in our case; however, our case diverged from this standard approach, as the patient, despite medical advice, declined wide local excision to avoid potential post-operative epiphora, given the lesion’s proximity to the punctum. Instead, he opted for close observation, making this case an interesting deviation from conventional management strategies. Most reported cases highlight the potential for recurrence and regional metastasis, necessitating vigilant follow-up. However, Chauhan et al. and Tak et al. reported in their cases that the standard 10 mm margin excision for PMC was not performed, as it was deemed unacceptable to the patient [8]. Tak et al. also documented a two-year follow-up period, during which no signs of recurrence were observed [12]. Remarkably, in our case, throughout the follow-up period, there was no evidence of recurrence, highlighting the indolent nature of the tumor and underscoring the importance of individualized patient-centered decision-making in such rare cases and the unpredictable nature of this malignancy.

Overall, this case adds to the expanding body of literature on this rare entity, emphasizing the need for a high index of suspicion even in innocuous-looking eyelid conditions. The divergence from conventional management and the patient’s favorable outcome make this case a noteworthy contribution to existing studies on primary mucinous carcinoma of the eyelid.

## Conclusion

This case highlights the diagnostic enigma of PMC of the eyelid, a malignancy often mistaken for benign lesions. It also underscores the importance of individualized treatment planning, where oncologic safety must be weighed against functional outcomes. While wide local excision remains the preferred approach, in select cases where surgical morbidity is significant, carefully monitored observation may serve as an alternative strategy if patients are compliant with follow-ups. This case serves as a reminder that even the most innocuous-looking lesions may harbor unexpected malignancies, reinforcing the indispensable role of histopathological evaluation in all excised eyelid tumors.
